# Emerging Non-statin Treatment Options for Lowering Low-Density Lipoprotein Cholesterol

**DOI:** 10.3389/fcvm.2021.789931

**Published:** 2021-11-17

**Authors:** Chandni Bardolia, Nishita Shah Amin, Jacques Turgeon

**Affiliations:** ^1^Office of Translational Research and Residency Programs, Tabula Rasa HealthCare, Moorestown, NJ, United States; ^2^Precision Pharmacotherapy Research and Development Institute, Tabula Rasa HealthCare, Lake Nona, FL, United States; ^3^Faculty of Pharmacy, Université de Montréal, Montréal, QC, Canada

**Keywords:** ezetimibe, PCSK9 inhibitors, alirocumab, evolocumab, bempedoic acid, inclisiran

## Abstract

Low-density lipoprotein cholesterol (LDL-C) is a modifiable risk factor for the development of atherosclerotic cardiovascular disease. Statins have been the gold standard for managing cholesterol levels and reducing the risks associated with atherosclerotic cardiovascular disease; however, many patients do not achieve their cholesterol goals or are unable to tolerate this drug class due to adverse drug events. Recent studies of non-statin cholesterol lowering drugs (i.e., ezetimibe, PCSK9 inhibitors) have demonstrated cardiovascular benefits; and new drugs [i.e., bempedoic acid (BDA), inclisiran] have produced promising results in pre-clinical and clinical outcome trials. This narrative review aims to discuss the place in therapy of ezetimibe, PCSK9 inhibitors, BDA, and inclisiran and describe their relative pharmacokinetic (PK) profiles, efficacy and safety as monotherapy and combination therapy, and cardiovascular benefit(s) when used for hypercholesterolemia.

## Introduction

Management of cholesterol, particularly low-density lipoprotein cholesterol (LDL-C) reduction, has been cited as one of the most reliable and achievable modifiable risk factors to reduce morbidity and mortality related to cardiovascular disease (CVD) (1). Together with lifestyle interventions (e.g., diet, exercise), HMG-CoA reductase inhibitors, commonly referred to as “statins”, are the gold standard for the management of cholesterol. Despite the efficacy and the widespread use of statins (2), patients are often not adherent to therapy and may discontinue these drugs due to statin-associated muscle symptoms (SAMSs) or fear of other adverse drug effects (ADEs) (1). One such ADE includes new-onset diabetes mellitus. Data currently suggests a 10–45% increase in risk of new-onset diabetes mellitus in statin users vs. non-statin users (3). Adverse drug effects like these and others (e.g., neurocognitive effects) are one of the drivers for the development of newer non-statin cholesterol-lowering medications.

Since the 1960's, pharmaceutical companies have developed drugs with unique mechanisms of action to manage cholesterol. Fibric acid derivatives (e.g., clofibrate, fenofibrate, gemfibrozil) are peroxisome proliferator-activated receptor alpha (PPAR-alpha) agonists. Binding to PPAR-alpha results in a decrease in triglycerides, an increase in high-density lipoprotein cholesterol (HDL-C), and only a modest decrease in LDL-C (4). Niacins, another class of non-statin medications, modulate lipolysis in adipose tissues but exhibit some tolerability and side-effect issues (5). Bile acid sequestrants/resins (e.g., cholestyramine, colesevelam) bind bile acids in the intestine, and excrete the complex in the feces preventing their reabsorption (6). Tolerability and dosage formulations tend to be limiting factors for the use of bile acid sequestrants (6). Ezetimibe, a cholesterol absorption inhibitor, is generally well-tolerated and demonstrates modest cardiovascular (CV) benefits (7, 8).

Proprotein convertase subtilisin/kexin type 9 (PCSK9) inhibitors and bempedoic acid (BDA) are some of the newer non-statin medications available within the United States (US) for the management of cholesterol. While PCSK9 inhibitors have published CV outcomes trials (9–12), BDA outcome trials are lacking at this time. Inclisiran, a small interfering RNA (siRNA) therapy, is another non-statin medication under investigation for cholesterol management. This narrative review aims to discuss the place in therapy of ezetimibe, PCSK9 inhibitors, BDA, and inclisiran (Table 1) and describe their relative pharmacokinetic (PK) profiles, efficacy and safety as monotherapy and combination therapy, and CV benefit(s) when used for hypercholesterolemia.

**Table 1:** Currently available and investigational non-statin medications for hypercholesterolemia.

**Drug name**	**Mechanism of action**	**Dosage**	**Frequency**	**Route of administration**	**LDL-C lowering (%)**	**Adverse drug effects**
Ezetimibe	Inhibit sterol transporter, NPC1L1	10 mg	Daily	Oral	−18.0	Diarrhea, arthralgia, upper respiratory tract symptoms
Alirocumab	Inhibit PCSK9 from binding to the LDL-C receptors	75 mg/ml 150 mg/ml	Q2W Q4W	Subcutaneous	−47.0 to −57.0	Injection site reactions, nasophary-ngitis, flu-like symptoms
Evolocumab		140 mg/ml 420 mg/ml	Q2W Q4W	Subcutaneous	−53.0	
Bempedoic acid	Inhibits ATP citrate lyase	180 mg	Daily	Oral	−21.4	Gout, throm-bocythemia, leukopenia, upper respiratory tract symptoms
Inclisiran	Inhibits the production of PCSK9 and blocks PCSK9 from binding to LDL-C receptors	300 mg/ml	Twice yearly	Subcutaneous	−29.9 to −46.4	Injection site reactions

## Role of Non-statin Therapies—A Brief History

### 2013 ACC/AHA Guideline on the Treatment of Blood Cholesterol

For the first time, in 2013, the American College of Cardiology (ACC) and American Heart Association (AHA) released lipid guidelines that strayed away from treating lipid values. Instead, they recommend primary or secondary prevention treatment based on the presence of a prior CV event or risk factor (e.g., heart disease, age, angina, peripheral artery disease) alongside lifestyle interventions (e.g., diet, exercise) (13). The primary goal of the 2013 guidelines was to identify and effectively manage the risk of atherosclerotic cardiovascular disease (ASCVD) (13).

While the 2013 guidelines greatly influenced medical practice, they included minimal guidance on the use of available non-statin therapies (i.e., fibric acid derivatives, niacin, bile acid sequestrants/resins, ezetimibe). The ACC/AHA panel recommended that clinicians consider adding an Food and Drug Administration (FDA)-approved non-statin medication in the following high-risk patients (13):

- Individuals who have a less-than-anticipated response to statins.
- Individuals who are unable to tolerate a less-than-recommended intensity of a statin.
- Individuals who are completely statin intolerant.

When selecting a non-statin therapy, the panel recommended that clinicians initiate a drug that has shown “ASCVD risk reduction benefits that outweigh the potential for adverse effects and drug-drug interactions (DDI)…” (13). At the time this guideline was published, none of the available non-statin demonstrated significant ASCVD benefits.

### 2016 Expert Consensus Decision Pathway on the Role of Non-statin Therapy

Along with lifestyle interventions for all patient groups, the 2016 ACC expert consensus designated ezetimibe as the non-statin therapy of choice to be initiated in clinically stable ASCVD patients requiring additional LDL-C lowering. This decision was based off the Improved Reduction of Outcomes: Vytorin Efficacy International Trial (IMPROVE-IT), discussed below. The 2016 consensus report indicated that PCSK9 inhibitors and bile acid sequestrants/resins were considered alternatives to ezetimibe. It also advised against routine use of niacin, due to lack of evidence for benefit (6).

### 2018 ACC/AHA Guideline on the Management of Blood Cholesterol

New guidelines were published in 2018 stating that non-statin therapy should be considered as secondary prevention treatment options for patients with a very high-risk of ASCVD events, for patients with statin intolerances, or for patients in whom a statin alone does not lower LDL-C sufficiently (<70 mg/dl). The 2018 guidelines cite ezetimibe, bile acid sequestrants/resins, and PCSK9 inhibitors as the non-statin LDL-C lowering drugs of choice. And per the 2018 guidelines, fibric acid and niacin derivatives should be reserved for individuals with increased triglyceride levels. Ultimately, the 2018 guidelines determined that more data are required to demonstrate the full benefit provided by non-statin medications (14).

## Ezetimibe

**Figure 1:**
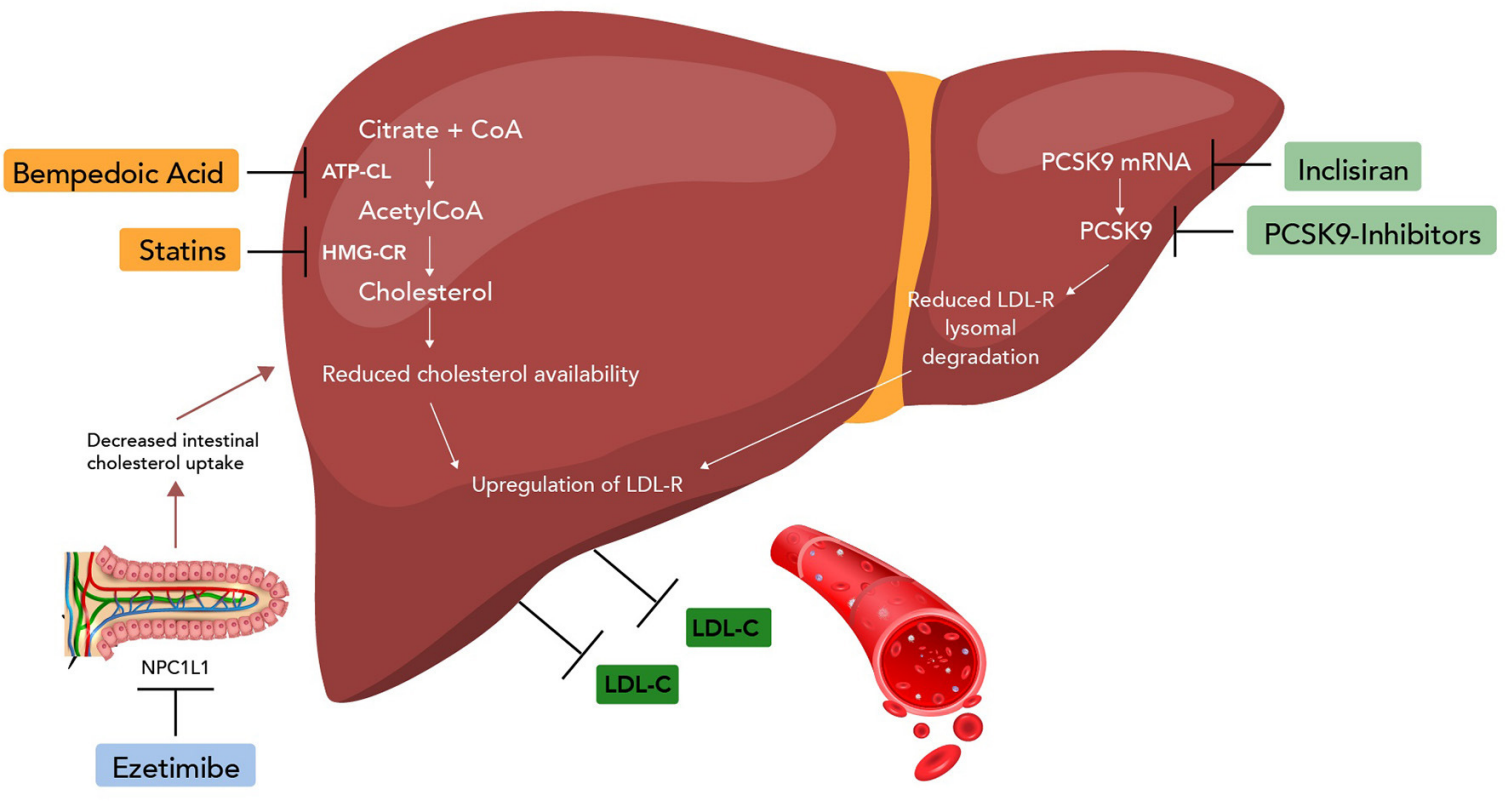
Mechanism of non-statin drugs (ezetimibe, PCSK9 inhibitors, bempedoic acid, and inclisiran). CoA, coenzyme A; ATP-CL, ATP citrate lyase; HMG-CR, HMG-CoA reductase; NPC1L1, Niemann-Pick C1-Like 1; mRNA, messenger RNA.

Ezetimibe is the only drug within the class of selective cholesterol absorption inhibitors. The 2018 ACC/AHA guidelines named ezetimibe as one of the designated non-statin alternatives and adjunct therapy options in patients at high-risk of ASCVD events (14). Prior to IMPROVE-IT, the lack of CV benefits limited the use of this medication; however, ezetimibe is now the second-line treatment option of choice for the management of hypercholesterolemia (14). Ezetimibe inhibits the absorption of cholesterol by targeting a sterol transporter called Niemann-Pick C1-Like 1 (NPC1L1; *SLC65A2*), which is localized at the brush border cells of the small intestine and involved in the uptake of cholesterol (15) (Figure 1). Inhibition of this transporter decreases the delivery of cholesterol into the mesenteric veins, hence to the liver, and ultimately increases the clearance of cholesterol from the blood (15).

### Ezetimibe: Pharmacokinetics

Upon oral administration, ezetimibe is absorbed and conjugated to a pharmacologically active phenolic glucuronide, ezetimibe-glucuronide (15, 16). The active metabolite is at least as potent as ezetimibe (17) and both are highly bound (90%) to plasma proteins, with a half-life of about 22 h (15, 16). Approximately 70% of the dose is found in feces as ezetimibe and nearly 10% is found in urine as ezetimibe-glucuronide (15, 16).

Studies indicate that the absorption of ezetimibe differs between younger adults (18–45 years of age) and older adults (>65 years of age) (15). The area under the curve (AUC; provides insight into the body's exposure to a drug) and the maximum concentration (Cmax) for ezetimibe were approximately 1.3- and 2-fold greater, respectively, in older adults compared to younger adults. The higher AUC indicates that older adults have greater exposure to ezetimibe and the higher Cmax indicates potential differences in absorption and/or clearance of ezetimibe in the older cohort. Though a higher Cmax and AUC were observed, the dose-response curves for LDL-C reductions between groups were not significantly different (15). Since no statistically significant dose-related toxicities were observed between the younger and older adult groups, dose adjustments are not warranted in older adult patients (15). Additionally, the presence of hepatic disease does not significantly affect the extent of ezetimibe conjugation, or increase its half-life (15). The presence of renal disease (creatinine clearance 10–29 ml/min/1.73 m^2^) results in a 50% higher exposure to ezetimibe (15). However, since the efficacy of ezetimibe is dose-related and not plasma concentration-related, the increased exposure to ezetimibe for patients with renal disease is deemed clinically insignificant. These latter findings indicate that no dosage adjustments are required for individuals with hepatic or renal impairment.

### Ezetimibe: Clinical Trials

Monotherapy with ezetimibe has demonstrated a mean percentage reduction of 18% in LDL-C from baseline when compared to placebo (18, 19). Pandor et al., revealed that ezetimibe monotherapy also improved total cholesterol (−13.46%, 95% CI: −14.22 to −12.70), HDL-C (3.00%, 95% CI: 2.06–3.94), and triglyceride concentrations (−8.06%, 95% CI: −10.92 to −5.20) when compared to placebo (19). LDL-C reductions observed with ezetimibe suggest that the drug may show at least partial efficacy for individuals unable to tolerate statins who require modest LDL-C reductions.

Though evidence dictates that ezetimibe monotherapy can reduce LDL-C levels, it is frequently used as an adjunct medication. In fact, the majority of studies involving ezetimibe are conducted in combination with various statin medications due, in part, to their complementary mechanisms of action. Co-administration of ezetimibe with a statin has been proven to result in additional LDL-C reductions, compared to monotherapy with either medication. Specific incremental reductions from baseline levels were 14, 12, 14, and 15% for ezetimibe administered with simvastatin, atorvastatin, pravastatin, and lovastatin, respectively, compared to statin monotherapy (*p* < 0.01) (20–23). The additional LDL-C lowering effects seen with ezetimibe in combination with low-dose statin therapy was shown to be equivalent to the LDL-C lowering effects of a high-dose statin (20–23). This finding may be beneficial for patients experiencing SAMSs with moderate or high-intensity statin doses.

For patients who do not tolerate statins, fenofibrate may be added to ezetimibe. Pandor et al. demonstrated that individuals receiving combination therapy or monotherapy with fenofibrate or ezetimibe achieved mean changes in LDL-C of −24.2% (combination therapy), −16.0% (fenofibrate), and −17.4% (ezetimibe) (24). As expected, ADEs were higher in the combination group compared to each monotherapy group; however, none of the ADEs were noted to be clinically significant (24). While this study offered fenofibrate as a potential adjunct to ezetimibe, neither medication had available CV outcomes data at the time of publication. This may have been a limiting factor in the widespread use of the combination.

Two studies, the Simvastatin and Ezetimibe in Aortic Stenosis (SEAS) and the Study of Heart and Renal Protection (SHARP), assessed efficacy, safety, and tolerability of adding ezetimibe to simvastatin in patients with aortic stenosis and chronic kidney disease (CKD), respectively (Table 2) (8, 25). A major limitation of these studies is that neither compared the impact of the combination to simvastatin alone; both trials compared the combination of ezetimibe and simvastatin to placebo. The SEAS trial concluded that the combination of ezetimibe and simvastatin did not reduce major adverse cardiovascular events (MACE) compared to placebo (95% CI: 0.83–1.12, *P* = 0.59) (8). The reason for this unexpected result can be explained, at least partially, by the selection of patients. The authors excluded individuals at high-risk of CV events (e.g., patients with diabetes, heart failure), which would reduce the number of CV events during the follow-up period. If the inherent risk of the included participants is low, the likelihood of treatment making a difference in CV outcomes would also be low. Conversely, the SHARP trial demonstrated a decrease in the incidence of MACE with the same combination (25).

**Table 2:** Landmark trials for ezetimibe.

	**SEAS**	**SHARP**	**IMPROVE-IT**
Year	2008	2011	2015
Duration	~52 months	48 months	84 months
Sample size	1,873	9,270	18,144
Study population	Individual between the ages of 45 and 85 years with asymptomatic, mild-to-moderate aortic-valve stenosis	Individuals with CKD and no history of MI or coronary revascularization	High-risk individuals with a recent history of ACS
Baseline LDL-C	139 mg/dl	108 mg/dl	93.8 mg/dl
Intervention(s)	Ezetimibe + simvastatin or placebo	Ezetimibe + simvastatin or placebo	Ezetimibe + simvastatin or placebo + simvastatin
Endpoint(s)	MACE (composite of CV death, aortic-valve replacement, congestive heart failure, non-fatal myocardial infarction, hospitalization for unstable angina, coronary-artery bypass grafting, percutaneous coronary intervention, or non-hemorrhagic stroke)	MACE (composite of myocardial infarction, coronary death, ischemic stroke, or any revascularization procedure)	MACE (composite of CV death, myocardial infarction, hospital admission for unstable angina, coronary revascularization at or 30 days after randomization, or stroke)
Outcome(s)	Combination of ezetimibe and simvastatin does not reduce MACE	Combination of ezetimibe and simvastatin resulted in a decrease in the incidence of MACE	Combination of ezetimibe and simvastatin resulted in a significantly lower risk of MACE
Progress	Completed	Completed	Completed

The IMPROVE-IT trial was a landmark study of ezetimibe on CV outcomes. The trial enrolled high-risk participants, after stabilization of acute coronary syndrome (ACS), to randomly receive either simvastatin plus ezetimibe or simvastatin plus placebo (26). Approximately 35% of participants in the combination group demonstrated a reduction in MACE, the primary endpoint of the study, compared to 33% in the simvastatin alone group (*p* = 0.02) (26). The number needed to treat was 50, which demonstrated a 2% absolute risk reduction in the primary endpoint (26). Results further indicated a 13% reduction in the incidence of MI and 21% reduction in the incidence of ischemic stroke, compared to the simvastatin only group (26). IMPROVE-IT results showed a modest reduction in ASCVD risk over a span of 7 years in patients post-ACS (26). Several studies following IMPROVE-IT have found that the benefits of concomitant ezetimibe and statin therapies were greatest in patients at high-risk of ASCVD (27–29). The ASCVD risk reduction seen with ezetimibe may not only be due to the LCL-C reduction, but also due to reduction of inflammation (30). Oh et al. have revealed that an ezetimibe/statin regimen (ezetimibe 10 mg/simvastatin 10 mg and ezetimibe 10 mg/rosuvastatin 5 mg) reduces carotid atherosclerotic plaque inflammation to a similar extent compared to statin monotherapy (rosuvastatin 10 mg and rosuvastatin 20 mg) (30). Masson et al. support this finding by demonstrating the addition of ezetimibe to statin therapy led to a reduction of coronary atherosclerosis (31).

### Ezetimibe: Safety Information

Though ezetimibe is regarded as a safe and effective treatment option today, the SEAS trial cast negative light on this medication. Safety analysis of the SEAS trial indicated that ezetimibe administration might have been associated with an increased incidence of cancer (8). Specifically, there were 101 new cancer diagnoses in the simvastatin plus ezetimibe group vs. 65 new diagnoses in the placebo group. This translated to a 50% increase in the incidence of cancer with ezetimibe (8). Shortly after IMPROVE-IT results were published, a meta-analysis of the participants in the SHARP trial and IMPROVE-IT found no significant difference in the incidence of cancer between combination and monotherapy groups (32). ACC/AHA expert consensus states that the risk of cancer should not deter physicians from prescribing ezetimibe.

Ezetimibe neither influences nor is influenced by the activity of CYP450 enzymes (15)—thus, minimal clinically significant PK interactions exist with ezetimibe (33). Ezetimibe combination trials indicated that there were no significant PK interactions with atorvastatin, simvastatin, pravastatin, and lovastatin (20–23). Pharmacokinetic studies of ezetimibe with gemfibrozil and fenofibrate resulted in a non-significant increase in the oral bioavailability of ezetimibe by a factor of 1.7 and 1.5, respectively (15). Cholestyramine and ezetimibe should be administered several hours apart due to a significant decrease in ezetimibe oral bioavailability (15).

### Ezetimibe: Conclusion

Though not yet approved for secondary prevention of ASCVD, studies indicate that ezetimibe monotherapy is an effective treatment option that has been shown to reduce LDL-C levels by 15–20% from baseline. While the efficacy of ezetimibe is independent of a statin, its LDL-C lowering effects and risk reduction for MACE are additive when combined with a statin. Patients at high risk of ASCVD benefited the most from the combination. Ezetimibe has been shown to have a tolerable safety profile both alone and in combination with other lipid lowering medications.

## Proprotein Convertase Subtilisin/Kexin Type 9 Inhibitors

Proprotein convertase subtilisin/kexin type 9 (PCSK9) is a protein that plays an important role in LDL-C regulation by binding to LDL receptors on hepatocytes (34). The main function of LDL receptors is to remove LDL-C from the circulation and prevent LDL-C deposit in peripheral tissues. The binding of PCSK9 to LDL receptors causes degradation of the receptors through intracellular pathways (34). Typically, LDL receptors would be recycled via endosomes; however, excess PCSK9 downregulates these receptors resulting in higher levels of circulating LDL-C (6, 14, 34). PCSK9 inhibitors are monoclonal antibodies that bind to the PCSK9 protein and prevent these proteins from binding to the LDL receptors, increasing receptor recycling, and permitting the uptake of circulating LDL-C.

The FDA approved the first PCSK9 inhibitor, alirocumab, in July 2015, shortly followed by evolocumab in August 2015. Initially, both were only approved for the treatment of hyperlipidemia in conjunction with statins. Presently, both PCSK9 inhibitors have received approval for monotherapy use, for homozygous familial hypercholesterolemia (HoFH), and for secondary prevention of CV events. Bococizumab would have been the third PCSK9 inhibitor and was undergoing multiple clinical trials; however, the trials were discontinued due to a higher level of immunogenicity and injection-site reactions compared to the currently available PCSK9 inhibitors (35). As such, the drug was withdrawn from development.

### PCSK9 Inhibitors: Pharmacokinetics

Following the subcutaneous delivery of a PCSK9 inhibitor, the drug is rapidly absorbed (36). Within days, LDL-C reductions of approximately 60% from the baseline value is observed and the effect is sustained for about 2 weeks at lower doses (36). Therefore, the dosing schedule is every 2 weeks for both PCSK9 inhibitors (37). PCSK9 inhibitor use results in concentration-dependent decreases in free PCSK9 and LDL-C levels until saturation of PCSK9 binding is reached (37). Once PCSK9 binding is saturated, further increases in alirocumab or evolocumab concentrations do not provide additional LDL-C reductions; however, higher concentrations do extend the duration of the LDL-C-lowering effect, which has allowed for extended dosing schedules (i.e., injections every 4 weeks) (37).

The absolute bioavailability of alirocumab and evolocumab is approximately 85 and 72%, respectively. Peak concentrations of alirocumab are achieved within 3–7 days (37) and 3–4 days (38) for evolocumab. Steady state concentrations are achieved after two to three doses of each medication (37). The volume of distribution for both drugs is approximately 0.05 L/kg (37). Alirocumab has a half-life of 17–20 days (37) and evolocumab has a half-life of 11–17 days (38).

### PCSK9 Inhibitors: Alirocumab Clinical Trials

Several trials were conducted that demonstrated the efficacy of alirocumab as monotherapy and in combination with other lipid lowering drugs. One of these trials compared ezetimibe monotherapy with various dosing regimens of alirocumab monotherapy (39). After 12 weeks of therapy, a 48% reduction in LDL-C from baseline was observed with 75 mg of alirocumab given every 2 weeks vs. a 20% reduction in the ezetimibe monotherapy group (39). Alirocumab resulted in substantial LDL-C reductions, which were maintained through the study period (39). Other studies demonstrated that alirocumab 150 mg every 4 weeks resulted in LDL-C reductions of 47–57% in combination with lifestyle modifications (40, 41). Alirocumab dosed at 300 mg every 4 weeks demonstrated reductions in LDL-C that were comparable to those observed with alirocumab 75 mg every 2 weeks (42). In patients with moderate-to-high CV risk who had reported intolerances to two or more statins in the past, alirocumab use resulted in LDL-C reductions of 45% from baseline, compared with a reduction of 14.6% for ezetimibe (43). While these trials did not report on CV outcomes, they set the stage for the CV outcomes trial with alirocumab.

The ODYSSEY OUTCOMES (Table 3) trial demonstrated that alirocumab, when added to a high-intensity or maximally tolerated statin, reduced the risk of recurrent ischemic cardiovascular events in patients with ACS (9, 12). Similar LDL-C reductions were observed in this study as previous alirocumab studies; however, MACE was significantly reduced for alirocumab vs. placebo (9.5 vs. 11.1%, HR 0.85, 95% CI 0.78–0.93, *p* < 0.001) (12). The authors of the ODYSSEY OUTCOMES trial noted that patients with a baseline LDL-C of >100 mg/dl yielded the most benefits from the alirocumab regimen (12), suggesting this population can be considered for such a regimen. Szarek et al. conducted a sub-analysis of the ODYSSEY OUTCOMES trial to determine the extent to which alirocumab reduced CV events and all-cause deaths (9). They demonstrated that the total number of non-fatal CV events and deaths prevented with alirocumab was twice the number of first events prevented (9). When parsing out the components of the composite endpoint, the use of alirocumab demonstrated reductions in non-fatal MIs, stroke, unstable angina, and all-cause mortality (9). The ODYSSEY OUTCOMES trial provided evidence that alirocumab has additive effects on both LDL-C reductions and CV benefits when combined with high-intensity statins.

**Table 3:** Landmark trials for PCSK9 inhibitors.

	**Odyssey outcomes**	**Mendel-2**	**Guass-2**	**Descartes**	**Fourier**
Year	2019	2014	2014	2014	2017
Duration	2.8 years	12 weeks	12 weeks	52 weeks	2.2 years
Sample size	18,924	615	370	901	27,564
Study population	Participants with a history of ACS, LDL-C level of at least 70 mg/dl, HD-C level of at least 100 mg/dl, or apolipoprotein B level at least 80 mg/dl	Participants unable to tolerate statins with a 10-year Framingham CHD risk scores ≤10%	Participants unable to tolerate ≥2 statins	Participants with LDL-C of 75 mg/dl or higher	Participants with ASCVD prescribed an optimized lipid-lowering regimen with LDL-C of 70 mg/dl or higher
Baseline LDL-C	86–92 mg/dl	~140 mg/dl	192–195 mg/dl	94–120 mg/dl	92 mg/dl
Intervention(s)	Alirocumab ± high-intensity statin or placebo ± high-intensity statin	Evolocumab or placebo + blinded ezetimibe	Evolocumab or placebo + blinded ezetimibe	Evolocumab or placebo + diet ± atorvastatin ± ezetimibe	Evolocumab or placebo + participants' optimized regimen
Endpoint(s)	MACE (composite of CHD-death, death, non-fatal MI, fatal and non-fatal ischemic stroke, or unstable angina requiring hospitalization)	Percent change from baseline in LDL-C level averaged at weeks 10 and 12 and at week 12	Percent change from baseline in LDL-C level averaged at weeks 10 and 12 and at week 12	Percent change from baseline in LDL-C at 52 weeks	MACE (composite of CV death, MI, stroke, hospitalization for unstable angina, or coronary revascularization)
Outcome(s)	MACE was significantly reduced in the alirocumab group compared to placebo (relative risk reduction of 15%)	Evolocumab reduced LDL-C from baseline by 55–57% more than placebo and 38–40% more than ezetimibe	Evolocumab reduced LDL-C from baseline by 53–56% more than placebo and 37–39% more than ezetimibe	Evolocumab reduced LDL-C from baseline by 57% more than placebo	Evolocumab significantly reduced the risk of MACE (1,344 patients [9.8%] vs. 1,563 patients [11.3%] compared to placebo)
Progress	Completed	Completed	Completed	Completed	Completed

In addition to the results observed in the ODYSEEY OUTCOMES trial, pooled sub-analyses of various ODYSSEY trials with alirocumab have continued to demonstrate benefits in high risk populations. Kereiakes et al. revealed that alirocumab improved the lipid/lipoprotein profile of high-risk patients with ASCVD, with or without prior revascularization (44). Significant reductions from baseline to week 24 in apoB, non-HDL-C, and lipoprotein a levels following alirocumab treatment were observed compared with control (placebo/ezetimibe) (44). Similarly, Vallejo-Vaz et al. demonstrated that patients with diabetes, CKD, or polyvascular disease appeared to yield greater absolute CV benefits from the additional LDL-C lowering with alirocumab than those without these chronic conditions (45).

### PCSK9 Inhibitors: Evolocumab Clinical Trials

Several evolocumab studies have been conducted comparing it to placebo and ezetimibe in statin-intolerant patients (Table 3). The Monoclonal Antibody Against PCSK9 to Reduce Elevated LDL-C in Subjects Currently Not Receiving Drug Therapy for Easing Lipid Levels-2 (MENDEL-2) (46) and Goal Achievement After Utilizing an Anti-PCSK9 Antibody in Statin Intolerant Subjects-2 (GAUSS-2) (47) trials included patients who were intolerant to two different statin medications. Between the two trials, approximately 1,000 participants were included and both studies revealed evolocumab resulted in greater LDL-C reductions from baseline when compared to placebo and ezetimibe. The Durable Effect of PCSK9 Antibody Compared with Placebo Study (DESCARTES) compared evolocumab monotherapy to low and high-dose atorvastatin with and without ezetimibe (48). While all treatment arms significantly reduced LDL-C levels in patients, those individuals utilizing low-dose atorvastatin plus evolocumab achieved a higher percentage reduction in LDL-C from baseline, compared to those taking high-dose atorvastatin plus evolocumab; this result was found to be statistically significant (*P* < 0.001) (48).

The Further Cardiovascular Outcomes Research with PCSK9 Inhibition in Subjects with Elevated Risk (FOURIER) trial demonstrated that evolocumab decreased LDL-C and major CV events (11). At 22 months, the use of evolocumab was associated with a 15% reduction in risk of MACE (11). Individual components of the composite endpoints were also associated with reduced risk, namely MI, stroke, and coronary revascularization. The study results demonstrated improved risk reduction with duration of evolocumab therapy from 12% the first year to 19% after 1 year (11). The addition of evolocumab to patients' regimens prevented 22 first CV events and 52 CV events for every 1,000 patients treated for 3 years (10). In this study, evolocumab did not significantly affect rates of hospitalizations, cardiovascular death, or death from any cause; as such, unlike the OYDESSY OUTCOMES trial, the FOURIER trial did not demonstrate cardiovascular mortality benefits with evolocumab.

Several sub-analyses of the FOURIER trial were conducted to investigate the safety and efficacy of evolocumab in patients with peripheral artery disease, CKD, and metabolic syndrome (49–51). Approximately 13% of the patients included in the FOURIER trial had peripheral artery disease and less than half had no prior MI or stroke. The addition of evolocumab to standard therapy significantly reduced the primary composite endpoint of MACE and reduced the risk of major adverse limb events (i.e., acute limb ischemia, major amputation, or urgent peripheral revascularization for ischemia) (50). The sub-analysis of patients with CKD indicated similar LDL-C reductions across all stages of CKD (49). Relative risk reduction of MACE was also similar in individuals with preserved kidney function; however, the absolute risk reduction of MACE was greater in the individuals with more advanced CKD, indicating a potential benefit of evolocumab for this specific patient population (49). Similar to patients with peripheral artery disease and CKD, patients with metabolic syndrome achieved clinically significant reductions in LDL-C and a reduction in the risk of CV events (51). These findings indicate that evolocumab can offer certain high-risk populations additional risk reduction via robust LDL-C reductions.

In addition to the LDL-C lowering demonstrated by both PCSK9 inhibitors, CV outcomes trials have demonstrated a reduced risk for MACE. While this may be due to the reduction in LDL-C, it may also be due, in part, to the reduction of coronary atherosclerosis (31). Similar to ezetimibe, PCSK9 inhibitors, in combination with a statin, have led to a regression in total atheroma volume (31, 52). Additionally, the potential anti-inflammatory effects of PCSK9 inhibitors, discussed further in the next section, may also contribute to the ASCVD risk reduction seen with this class of medication (53).

### PCSK9 Inhibitors: Safety Information

PCSK9 inhibitors are generally considered safe and well-tolerated. The longest safety trial for PCSK9 inhibitors is approximately 5 years, and serious ADEs were uncommon. In fact, the rate of ADEs with PCSK9 inhibitors was similar to placebo (54). The most common ADE among users of PCSK9 inhibitors is injection site reactions (11, 46, 48, 55). Studies for PCSK9 inhibitors also monitored for new-onset diabetes and neurocognitive effects, two ADEs that have been associated with long-term statin use. To date, no clinical trials have demonstrated an increase rate of diabetes with PCSK9 inhibitor use (11, 56, 57).

Robinson et al. monitored for neurocognitive events with alirocumab and indicated that neurocognitive disorders were more frequent with the alirocumab group compared to the placebo group; however, the neurologic events were linked to immunologic and inflammatory causes (56). For evolocumab, a FOURIER sub-analysis studied the mean change from baseline score on the spatial working memory strategy index of executive function between patients who received the drug and those who received placebo (58). No significant differences in cognitive function test scores or in subjective self-assessments of everyday cognition were observed. Additionally, neither treatment arm improved or worsened executive function, working memory, episodic memory, or psychomotor speed (58). Sabatine et al. noted that pushing LDL-C levels below 20 mg/dl with either PCSK9 inhibitor therapy resulted in no signs of mental harm and no changes in self-reported cognition when compared to placebo (59). This confirms the previous notion of “lower is better” and tends to refute the J-curve concept associated with statins.

More recently, studies have been discussing the potential role of PCSK9 and the PCSK9 inhibitors in atherosclerosis. The PCSK9 protein has been associated with various inflammatory markers including white blood cells, C-reactive protein, and fibrinogen in individuals with ACS and coronary artery disease (53). Evidence also shows that PCSK9 can enhance the production of pro-inflammatory cytokines that play an important role in atherosclerosis plaque inflammation (53). PCSK9 inhibitors can work to counter-act some of these effects; however, human studies are lacking to demonstrate direct benefits of PCSK9 inhibitors on the previously mentioned inflammatory markers and pro-inflammatory cytokines (53).

Since PCSK9 inhibitors are injectable monoclonal antibodies, there are very few known DDIs. Concomitant statin and PCSK9 inhibitor use has demonstrated a reduction in the half-life of alirocumab, as well as a 20% reduction in Cmax and AUC of evolocumab (38). The reduction in these parameters is primarily due to an increase in the PCSK9 protein levels (48, 60) which leads to lower concentrations of unbound antibodies (38). The effect of these interactions is not clinically significant and dosing adjustments are not warranted.

### PCSK9 Inhibitors: Conclusion

A PCSK9 inhibitor should be selected based on the patient's goals of therapy, current LDL-C levels, and CV risk. The lower LDL-C levels achieved with either PCSK9 inhibitor are associated with CV risk reduction for patients deemed high risk. The defining difference between the two available PCSK9 inhibitors is mortality benefits, favoring alirocumab, at this time. Overall, the safety profile of PCSK9 inhibitors is favorable and the injections appear to be well-tolerated. Additionally, because the evidence indicates there are no signs of worsening cognition or new onset diabetes, PCSK9 inhibitors may be a good option for patients who are particularly concerned about these ADEs with long-term statin use. A limiting factor for the widespread adoption of PCSK9 inhibitors is cost. At this time, PCSK9 inhibitors are only proving to be cost-effective for high-risk ASCVD patients; however, in a patient who is unable to maintain therapy with statins, PCSK9 inhibitors may confer significant benefits (5–7, 31–41).

## Bempedoic Acid

Bempedoic acid (BDA) is a once daily prodrug that requires activation by the enzyme very-long-chain acyl-CoA synthetase A (61). Very-long-chain acyl-CoA synthetase A is an enzyme mainly expressed in the liver and kidney, but not expressed in skeletal muscle. The active metabolite of BDA, ESP15228, inhibits ATP citrate lyase, an enzyme upstream of HMG-CoA reductase (61). ATP citrate lyase is a vital enzyme in the cholesterol biosynthesis pathway and is responsible for the production of acetyl CoA from citrate (61). Inhibiting ATP citrate lyase prevents *de-novo* cholesterol synthesis in hepatocytes (61). By inhibiting cholesterol synthesis, BDA results in increased LDL-C receptor expression (61). Upregulation of these receptors results in increased clearance of LDL-C.

### Bempedoic Acid: Pharmacokinetics

BDA has a volume of distribution of 18 L. Both BDA and its active metabolite are highly (99.3 and 99.2%, respectively) plasma protein bound (62). BDA achieves maximum concentration within a median time of 3.5 h and steady state after approximately 7 days (62). The half-life of BDA is 15–24 h (62).

### Bempedoic Acid: Clinical Trials

The efficacy and safety of BDA has been evaluated by five CLEAR trials: Harmony, Wisdom, Serenity, Tranquility, and Outcomes (Table 4). The CLEAR Harmony (Assessment of the Long-Term Safety and Efficacy of Bempedoic Acid) and CLEAR Wisdom (Efficacy and Safety of Bempedoic Acid Added to Maximally Tolerated Statins in Patients With Hypercholesterolemia and High Cardiovascular Risk) trials evaluated the safety and efficacy of BDA in patients already on maximally tolerated statins. Both CLEAR Harmony and CLEAR Wisdom demonstrated BDA significantly reduced LDL-C levels at week 12 by an additional 16.5 and 15.1% respectively, compared to placebo at week 12 (63, 64). In both trials, BDA significantly reduced total cholesterol, ApoB, non-HDL-C, and high-sensitivity C-reactive protein (hsCRP) compared to placebo (63, 64). Though not powered to evaluate CV outcomes, the CLEAR Harmony trial noted a difference in the rates of MACE between the BDA arm (4.6%) and placebo arm (5.7%) (63).

**Table 4:** Landmark trials for bempedoic acid (BDA).

	**Harmony**	**Wisdom**	**Serenity**	**Tranquility**	**Outcomes**
Year	2019	2019	2019	2018	TBD
Duration	52 weeks	52 weeks	24 weeks	12 weeks	3.75 years
Sample size	2,230	779	345	269	14,014
Study population	Individuals with ASCVD, HeFH, or both receiving max tolerated statin	Individuals with ASCVD, HeFH, or both receiving max tolerated statin	Individuals with a history of statin intolerance to ≥2 statins	Individuals with a history of statin intolerance with no statin or low-dose statin	Individuals with a history of, or at high-risk for CVD with reported statin intolerance
Baseline LDL-C	102–103 mg/dl	120.4 mg/dl	157.6 mg/dl	127.6 mg/dl	–
Intervention(s)	BDA or placebo + background therapy	BDA or placebo + background therapy	BDA or placebo + background therapy	BDA or placebo + ezetimibe	BDA or placebo + background therapy
Endpoint(s)	Percentage change in LDL-C at week 12 of 52 weeks	Percentage change in LDL-C at week 12	Percentage change in LDL-C at week 12	Percentage change in LDL-C at week 12	MACE (composite of CV death, non-fatal MI, non-fatal stroke, or coronary revascularization)
Outcome(s)	BDA reduced LDL-C levels by 16.5% at week 12	BDA reduced LDL-C levels by 15.1% at week 12	BDA reduced LDL-C levels by 21.4% at week 12	BDA + ezetimibe reduced LDL-C levels by 28.5% at week 12	TBD^*^
Progress	Completed	Completed	Completed	Completed	Active NCT02993406

* *TBD, to be determined*.

The CLEAR Serenity (Evaluation of the Efficacy and Safety of Bempedoic Acid in Patients With Hyperlipidemia and Statin Intolerance) and Tranquility (Evaluation of the Efficacy and Safety of Bempedoic Acid as Add-on to Ezetimibe Therapy in Patients With Elevated LDL-C) trials studied the change in LDL-C in patients treated with low-dose statins due to their inability to tolerate high dose statins (65). The CLEAR Serenity trial mirrored LDL-C reductions observed in previous studies; however, there were two findings of particular interest in this study. CLEAR Serenity showed significantly less LDL-C reduction at week 12 for diabetic patients compared to non-diabetic patients (65). Additionally, MACE occurred in nine BDA users vs. zero patients in the placebo group (65). Of note, these findings were not seen in previous studies, nor were they replicated in the studies that followed. The CLEAR Tranquility trial measured the effects of adding BDA onto ezetimibe therapy and deemed it to be a beneficial add-on in patients unable to tolerate statins (66).

A CV outcomes trial, CLEAR Outcomes (Evaluation of Major Cardiovascular Events in Patients With, or at High Risk for, Cardiovascular Disease Who Are Statin Intolerant Treated With Bempedoic Acid or Placebo; NCT02993406) is underway with BDA, and results are expected to be released in 2023. The primary endpoint of the study is the effect of BDA on MACE. Though no study results to-date indicate whether BDA provides CV benefit(s), several trials note that BDA has resulted in significant reductions in hsCRP (63, 64, 66). Elevations in hsCRP have been linked to increased risk for coronary heart disease and adverse CV outcomes, therefore a reduction hsCRP may lead to positive CV outcomes.

### Bempedoic Acid: Safety Information

Phase three clinical trials indicate that BDA is safe and well-tolerated (66). Bempedoic acid has not been shown to cause myopathies to the same degree as statins. Although head-to-head trials have not been conducted to date (61), comparable rates of discontinuation and muscle-related ADEs between BDA and placebo have been reported (66). This may be due, in part, to the fact that very-long-chain acyl-CoA synthetase A, which is required for BDA activation, is not expressed in skeletal muscle (67). Elevations in uric acid were observed more frequently in individuals using BDA compared to placebo. However, upon further analysis, most individuals experiencing this ADE had either a history of gout or pre-study elevations of uric acid (63–66). The CLEAR Harmony trial noted BDA was associated with higher rates of new onset or worsening of diabetes, but the subsequent CLEAR trials did not substantiate these findings (63). The most commonly reported ADEs were nasopharyngitis, myalgia, upper respiratory tract infection, urinary tract infection, arthralgia, dizziness, muscle spasms, and diarrhea—all of which occurred with similar frequency in the BDA and placebo groups (63).

Limited information is available on DDIs involving BDA. *In vitro* studies indicate that co-administration of BDA with simvastatin or pravastatin resulted in increased concentrations of these statins (68). The exact mechanism for the interaction is not understood, but likely related to the glucuronide metabolite weakly inhibiting OATP1B1 at higher doses (e.g., 240 mg) (68). This interaction is problematic for two reasons: (1) inhibition of OATP1B1 increases plasma concentrations of statins resulting in an increased risk for statin-related myopathies; (2) inhibition of OATP1B1 reduces efficacy of statins since functional OATP1B1 transporters are required for statin transport to hepatocytes (the site of action for statins). Administration of simvastatin 40 mg with BDA 180 mg resulted in a 96% increase in simvastatin AUC (68). Administration of pravastatin 40 mg and BDA 240 mg resulted in a 99% increase in pravastatin AUC (68). Increases in the AUC of atorvastatin and rosuvastatin were also noted, however, the increases observed were deemed to be within the normal statin exposure range (68). Therefore, concomitant use of BDA with simvastatin doses >20 mg or pravastatin doses >40 mg is not recommended, and no dosage adjustments are required with atorvastatin or rosuvastatin (68). In addition to DDIs, BDA has a few drug-disease interactions that require attention. BDA has resulted in decreases in hemoglobin levels and leukocyte counts and increases in platelet counts (68). BDA use has also been associated with an increased risk of benign prostatic hyperplasia and tendon rupture (68), which may limit its use in certain populations.

### Bempedoic Acid: Conclusion

Bempedoic Acid is available within the US as 180 mg tablets. Also, due to its complimentary mechanism of action with ezetimibe, the two drugs are available as a single tablet combination (69). While further studies are required to establish CV benefits, BDA is effective for reducing LDL-C when used with dietary modifications and/or with other lipid-lowering therapies. The recent approval of BDA in the US presents it as another therapeutic option for patients diagnosed with hypercholesterolemia; however, its place in therapy will be better defined once results for the CLEAR Outcomes trial are published.

## Inclisiran

Inclisiran, an investigational cholesterol-lowering drug, is a long acting, double stranded, siRNA molecule (70). It inhibits the production of PCSK9 in the liver by cleaving messenger RNA required for PCSK9 production and lowers LDL-C levels by preventing the interaction of PCSK9 with LDL receptors (70). This action results in the upregulation of LDL receptors, thereby increasing LDL-C uptake and reducing circulating LDL-C (70).

### Inclisiran: Pharmacokinetics

Inclisiran reaches maximum plasma concentrations after 4 h (71). It has a short plasma half-life (5–10 h), which is not impacted by renal impairment; however, it is renally eliminated (71). After a single injection, the LDL-C effects of the drug are reversed at a rate of about 2% per month (70). As such, inclisiran is administered subcutaneously at day 0, 90, and then every 6 months thereafter.

### Inclisiran: Clinical Trials

Phase one studies demonstrated that the magnitude of LDL-C lowering with inclisiran was similar to that observed with high-intensity statins or PCSK9 inhibitors (72). The ORION-1 dose ranging study, which included participants on maximally tolerated statins, determined that a two-dose regimen of the drug provides the greatest LDL-C reductions with minimal ADEs, when compared to placebo (11 vs. 8%) (73).

While several ORION trials are being conducted, published results from phase two studies demonstrated the efficacy and safety of inclisiran (Table 5). The ORION-10 trial compared inclisiran with placebo in patients with ASCVD, while the ORION-11 trial enrolled patients with ASCVD risk equivalents (e.g., type two diabetes, familial hypercholesterolemia, or a 10-year risk of CV events of ≥20%) (70). Results for both studies were presented in a single publication by Ray et al. ORION-10 results indicated that twice-yearly injections of inclisiran reduced LDL-C by 56%, compared to an increase of 1% with placebo (70). ORION-11 results demonstrated a mean reduction in LDL-C in the inclisiran group to be 49%, compared to an increase in 4% in the placebo group (70). Both the ORION-10 and−11 included an exploratory CV endpoint, but the total number of CV events observed was too small to draw any meaningful clinical conclusion (70). Results from the ORION-4 trial may provide greater insight on the benefits of inclisiran on reducing ASCVD risk.

**Table 5:** Landmark trials for inclisiran.

	**ORION-10 and 11**	**ORION-3**	**ORION-4**	**ORION-8**
Year	2020	2022 anticipated	TBD	TBD
Duration	540 days	4 years^*^	5 years^*^	~3 years^*^
Sample size	3,178 (1,561 and 1,617, respectively)	490	15,000^*^	2,991^*^
Study population	Individuals with ASCVD (ORION-10) and ASCVD or ASCVD risk equivalents (ORION-11)	Individuals with ASCVD or ASCVD risk equivalents	Individuals with a history of ASCVD	Individuals with ASCVD, ASCVD risk equivalents, HeFH, or HoFH
Baseline LDL-C	104.7 mg/dl (ORION-10)	–	–	–
	105.5 mg/dl (ORION-11)			
Intervention(s)	Inclisiran or placebo	Inclisiran or evolocumab followed by inclisiran	Inclisiran or placebo	Inclisiran or placebo
Endpoint(s)	Percentage change in LDL-C from baseline to day 510 and percentage change in LDL-C from baseline after day 90 and up to day 540	Percentage change in LDL-C at day 210	MACE (composite of CHD-death, MI, fatal or non-fatal ischemic stroke, or urgent coronary revascularization)	Proportion of individuals achieving LDL-C targets <100 and <70 mg/dl
Outcome(s)	Inclisiran reduced LDL-C levels by 51.3% (ORION-10) and 45.8% (ORION-11) at day 510	TBD	TBD	TBD
Progress	Completed	Active	Active	Active
		NCT03060577	NCT03705234	NCT03814187

* *Estimate*.

ORION-4 (NCT03705234), a phase three study, is presently recruiting participants with pre-existing ASCVD and who are unable to achieve LDL-C goals. Expected to finish in 2024, this trial will elucidate inclisiran's effects on CV outcomes. There are projected to be 20 ORION trials with inclisiran. Of these, five will involve special populations (e.g., individuals with renal impairment, hepatic impairment) and focus on the PK of inclisiran. ORION-3 (NCT03060577) is a phase two, active comparator trial to assess the efficacy, safety, and tolerability of inclisiran and evolocumab given to participants with high CV risk and elevated LDL-C. ORION-8 (NCT03814187) is also an ongoing trial, that will assess the long-term efficacy and safety of inclisiran.

### Inclisiran: Safety Information

In the safety analysis of ORION-1, no ADEs related to inflammation, immune activation, or clinical immunogenicity were observed (74). Treatment-emergent ADEs and serious ADEs were similar between inclisiran and placebo in both the ORION-10 and−11 trials (70). There were no signs of liver, kidney, or muscle toxicity in any trial with inclisiran; however, injection site reactions were significantly more common with inclisiran compared to placebo (70, 75). There have been no reported DDIs with inclisiran and statins, or any other medications (70). Additionally, the safety of inclisiran has been tested in individuals with renal impairment and dose adjustments are not required in this subgroup of patients (71).

### Inclisiran: Conclusion

In December 2020, the FDA declined to approve inclisiran. The agency stated there were no concerns related to safety and efficacy, but were unable to grant the new drug application due to the inability to conduct a facility inspection (76). As the wait for FDA approval continues, the published ORION trials have demonstrated promising LDL-C reduction with inclisiran. The use of inclisiran in combination with maximally tolerated statins has resulted in an additional 50% reduction in LDL-C, and the twice-yearly administration offers an advantage over currently approved treatment options. Until the results for these studies are published and inclisiran receives FDA-approval, it will be difficult to ascertain its role in managing patients with hypercholesterolemia.

## Cost

Cost is often a contributing factor when selecting therapy for hypercholesterolemia. Of the discussed non-statin medications, only ezetimibe is available as a generic product within the US. Per the Cleveland Clinic, the approximate wholesale price (AWP) for ezetimibe is $840, annually (77). Since approval, both alirocumab and evolocumab have undergone drastic changes in price. Manufacturers of both drugs reduced list price by 60% and now both drugs are available at an AWP of $5,850, annually (78, 79). Approximate wholesale price for BDA is $4,750, annually (80). No cost information has been released for inclisiran.

In addition to yearly costs, the cost-effectiveness of these non-statin therapies will play a role in determining their uptake into clinical practice. Several cost-effectiveness studies have been conducted with ezetimibe and PCSK9 inhibitors. While most cost-effectiveness studies of ezetimibe are conducted in combination with statin therapy, Ara et al. evaluated the cost-effectiveness of long-term ezetimibe monotherapy in patients. For statin-intolerant individuals with CV disease, the authors estimated that ezetimibe monotherapy would prevent approximately 4.9% of non-fatal MIs, 1.1% of non-fatal strokes, and 3.7% of CV deaths-deeming it cost-effective compared to no treatment (81).

The majority of cost-effectiveness studies with PCSK9 inhibitors were conducted prior to their price reductions. Based on this data, PCSK9 inhibitors are not cost effective (82–85). One study indicated revascularization surgeries would have a more favorable cost profile compared to PCSK9 inhibitors (82). It was determined that heavy price reductions were required in order for both medications to be cost-effective at < $100,000 per quality-adjusted life-year (QALY) (85). One study demonstrated that, in the ASCVD population, adding PCSK9 inhibitors to statins may prevent 4.3 million occurrences of MACE; however, to be considered cost-effective, drug costs would need to be $4,536 per patient, annually, or less (85).

Recently, the Institute of Clinical and Economic Review announced plans to assess BDA, along with other non-statin medications, for clinical cost-effectiveness (86). Their full report is slated to be available by the end of 2021 (86); however, the Institute of Clinical and Economic Review's Midwest Public Advisory Council did publish a summary of the effectiveness and value of BDA and inclisiran for secondary prevention of ASCVD and heterozygous familial hypercholesterolemia in July 2021 (87). The council found that at current estimated pricing, BDA is unlikely to achieve cost-effectiveness thresholds of $100,000–$150,000 per QALY (87). For inclisiran to be considered cost-effective at threshold of $150,000 per QALY, it would have to be priced at $5,644 per year (87).

## Conclusion

The first statin medication was approved in in 1987; and, while statins have been the gold standard for hypercholesterolemia for decades now, many patients do not achieve their LDL-C goals or are unable to tolerate cholesterol-lowering medication due to ADEs. In the early 2000s, ezetimibe was approved for hypercholesterolemia, followed by PCSK9 inhibitors several years later, and BDA more recently. Ezetimibe and PCSK9 inhibitors have shown positive outcomes related to MACE in patients with clinical ASCVD; however, several clinical outcomes trials are still in progress for BDA and for the investigational drug, inclisiran. These non-statin therapies have all shown efficacy in reducing LDL-C as monotherapy and in combination therapy with statins or other non-statin medications. Safety profiles are generally favorable with these medications. Further studies and post-marketing trials will highlight additional ADEs and interactions for BDA and inclisiran. Given the current evidence and cost data for with non-statins, especially PCSK9 inhibitors and BDA, these medications should be reserved for high-risk patients (with ASCVD or risk equivalents) who require additional LDL-C reductions or those unable to tolerate high-intensity statins. With more novel medications being approved for hypercholesterolemia, patients and clinicians now have non-statin options for robust LDL-C reductions and CV benefits. Until a future iteration of the ACC/AHA guidelines recommend a place in therapy for these newer non-statins, they should be prescribed to patients on a case-to-case basis.

## Author Contributions

The idea for the article was conceptualized by JT. CB performed the literature search and analysis along with drafting the manuscript. JT and NA reviewed and critically revised the work. All authors contributed to the article and approved the submitted version.

## Conflict of Interest

CB, NA, and JT are employed by the company Tabula Rasa HealthCare. The handling Editor declared a shared affiliation, though no other collaboration, with one of the authors JT.

## Publisher's Note

All claims expressed in this article are solely those of the authors and do not necessarily represent those of their affiliated organizations, or those of the publisher, the editors and the reviewers. Any product that may be evaluated in this article, or claim that may be made by its manufacturer, is not guaranteed or endorsed by the publisher.
